# Association Between Changes in Muscle Strength and Risk of Depressive Symptoms Among Chinese Female College Students: A Prospective Cohort Study

**DOI:** 10.3389/fpubh.2021.616750

**Published:** 2021-04-08

**Authors:** Jianhua Cao, Fang Zhao, Zhongyu Ren

**Affiliations:** ^1^School of Physical Education and Health, Nanning Normal University, Nanning, China; ^2^Key Laboratory of Physical Fitness Evaluation and Motor Function Monitoring, College of Physical Education, General Administration of Sport of China, Southwest University, Chongqing, China

**Keywords:** depressive symptoms, muscle strength, Chinese college students, handgrip strength, incidence risk

## Abstract

Muscle strength can be a predictor of depressive symptoms among the elderly. We conducted a prospective study aiming to examine the association between change of handgrip strength and the incidence risk of depressive symptoms among Chinese female college students. Handgrip strength was used as a representative indicator of skeletal muscle strength, and a handheld digital smedley dynamometer was applied to measure handgrip strength. We also used the 20-item Zung self-rating depression scale to evaluate depressive status, and a score of ≥50 indicated moderate-to-severe depressive symptoms. During a 1-year follow-up period, the incidence of depressive symptoms is 10.7%. Multivariate logistic regressions analysis revealed that the multivariable-adjusted ORs (95% CI) of depressive symptoms for the categories of handgrip strength change was 1.00 (reference) for group 1, 0.57 (0.28, 1.19) for group 2, 0.41 (0.19, 0.89) for group 3 and 0.33 (0.11, 0.99) for group 4 (*p* = 0.018). This study indicated that change of handgrip strength level over one-year period is negatively associated with risk of depressive symptoms among Chinese female college students.

## Introduction

Depression, characterized by sadness or irritability and accompanied by at least several psychophysiological changes, such as disturbances in sleep, appetite, or sexual desire (1), is currently the most common mental disorder. According to World Health Organization statistics, ~350 million people suffered from depression worldwide (2). Moreover, depression not only contributes to 4.4% of disease burden (3), but is also identified as a leading cause of suicide (4). Thus, it is necessary to identify effective preventive factors for depression.

It is widely known that increased inflammatory secretion may play a significant role in etiopathogenetic of depression. Inflammatory cytokines have been suggested to alter neurotransmission (5), hippocampal neurogenesis (6), and stress-related hypothalamic-pituitary-adrenal (HPA) axis (7) and sympathetic system activation (8), which can cause changes in structure and function of the brain and subsequent development of depression (9). Skeletal muscle, a major secretory organ, can secrete and produce numerous proinflammatory cytokines, such as IL-6, IL-8, and IL-15 (10). Previous studies confirmed that weaker skeletal muscle strength led to increased serum proinflammatory cytokines among younger adult (11, 12). Based on these findings, it is reasonable to speculate that skeletal muscle could be associated with depressive symptoms.

Thus far, accumulated evidences has revealed a cross-sectional (13–15) and prospective (16–19) relationship between muscle strength and depressive symptoms among middle-aged and elderly adults. However, individuals frequently change their physical activity level over time, which subsequently affects their muscle strength level. Thus, previous studies examining the associations of baseline muscle strength level with risk of depressive symptoms cannot adequately explain whether consistently or transiently keeping a higher level of muscle strength, consistently or transiently, can prevent risk of depressive symptoms. Examining how changes in muscle strength level affect risk of depressive symptoms can provide a more complete understanding of the relationships between muscle strength level and depressive symptoms. Furthermore, evaluating the association of short-term changes in muscle strength level with the morbidity of depressive symptoms over time will help identify which components of the exercise program will be conducive in preventing morbidity of depressive symptoms. To our knowledge, no study has examined the association between changes in muscle strength level and risk of depressive symptoms. As half of all lifetime cases of depression start by the age of 14 and three quarters by the age of 24 (20), it is crucial for public health professionals to pay attention to depression of younger adults.

Therefore, this study aimed to examine whether or not muscle strength change is associated with risk of depressive symptoms during 1-year among Chinese college students.

## Materials and Methods

### Study Participants

The CNVCPFH study, which was an prospective cohort study, was carried out from 2018 to 2020 among 1094 college freshmen at the Chongqing Nursing Vocational College. A detailed study design has been provided previously (21). All 1,094 individuals participated in the annual physical fitness examination at baseline. We invited and recruited these 1,094 participants, who agreed to participate in this study, and obtained their written informed consent for analysis of their data from all college freshmen aged ≥16 years or from the legal guardians of participants aged <16 years. We excluded 121 participants with missing information on handgrip strength (*n* = 59), PA (*n* = 62) and male participants (*n* = 106). Ninety-three participants were also excluded due to existing depressive symptoms at baseline. During the 1-year follow-up period, we excluded 175 participants due to missing data on handgrip strength. Finally, the prospective study was composed of 599 female participants [mean 18.7, range (16–23) years].

### Power Calculations

Power calculations for the study sample size were done using the following formula:

$$\begin{array}{l} N={\left(\frac{u_{\alpha }\sqrt{2PQ}+u_{\beta }\sqrt{P_{1}\left(1-P_{1}\right)+P_{2}\left(1-P_{2}\right)}}{P_{1}-P_{2}}\right)}^{2} \end{array}$$

Where N = sample size

- P1 = experimental group; P2 = control group
- P = (P1+P2) / 2; Q = 1-P
- u_α_ = 0.05; u_β_ = 0.1

In a Chinese population-based national survey, the prevalence rate of depressive symptoms in a control group and experimental group is 32 and 15.7%, respectively (19). Based on these calculations, it was estimated that the minimum total sample size of 119 would adequately provide 90% power to detect a significant difference between groups.

### Assessment of Depressive Symptoms

This study used the Chinese version of the Zung self-rating depression scale (SDS), which is a self-reporting instrument used to evaluate depression severity (22). The SDS contains 20 questions (10 positively-worded and 10 negatively-worded questions) with 4 responses (none, a little of the time, most of the time, or all of the time). The 10 positively-worded questions were graded on a scale of one to four and the 10 negatively-worded questions were graded on a scale of four to one. All scores for the 20 item questionnaire were summed up to produce a total score; a higher score indicated severe depression severity. We used a SDS score of ≥50 to define mild and severe depressive symptoms. We also used Cronbach's α coefficients to describe the reliability of the Chinese version of the SDS. The Cronbach's α coefficients were 0.730 and 0.818, respectively, in 2018 and 2019, indicating that the SDS has a high internal consistency. The sensitivity and specificity of the SDS is 92.3 and 87.5%, respectively (23) and Youden index (sensitivity + specificity-1) is 0.798.

### Assessment of Handgrip Strength

We used a digital dynamometer (EH101; CAMRY, Guangdong, China) to assess handgrip strength level. To ensure that the handgrip strength was measured correctly and each participant was very familiar with measurement demands, a trained technician guided the participants, demonstrated each movement to the participants, and encouraged each participant to squeeze the dynamometer as hard as possible during the four measurements. The trained technicians asked each participant to stationarily stand, relax their arm and adjust the dynamometer width in order to make their hand comfortable. Each participant was asked to maintain shoulders back and shoulder abducted ~10°, arm straight down side, elbow fully extended, and wrist in neutral position. Each participant was told to squeeze the grip as hard as possible while exhaling and made four attempts using each hand with intervals of at least 30 s. The maximum value of four measurements represents muscle strength level. We conducted a test-retest for handgrip strength to evaluate measurement stability and found the intraclass correlation coefficient between the two assessments to be 0.737 [95% confidence interval (CI): 0.698, 0.771], which is considered highly stable. Change in handgrip strength at 1 year was divided into 4 categories according to ± 1SD: group 1 (≤-3.6), group 2 (−3.5, 0.1), group 3 (0.2, 3.8), and group 4 (≥3.9).

### Assessment of Relevant Covariates

Body mass index (BMI) was calculated by following formula: Weight/(height)^2^. Demographic variables [age (continuous variable)], smoking and drinking status (never, occasionally, or regularly), sleep duration (continuous variable), and quality (good or not good) were obtained from a self-reported questionnaire. Two self-reported questions was used to assess sleep duration and sleep quality: “How many hours of sleep did you usually get at night;” and sleep quality subscale: “During the past month, how would you rate your overall sleep quality?”. Previous study conducted a test-retest for these two items to evaluate measurement stability among Chinese population (including college students), and found the correlation coefficient between the two assessments to be 0.765 and 0.566, which are considered highly stable (24, 25). Physical activity levels were assessed using the Chinese short version of the International Physical Activity Questionnaire (IPAQ-C) (26). Intraclass correlation coefficient and spearman's rank correlation coefficient were used to evaluate the reliability and validity of the IPAQ-C, respectively (26). The intraclass correlation coefficient and spearman's rank correlation coefficient were 0.779 and 0.598, respectively (26), indicating that the IPAQ-C had good reliability and validity (26). The short version of IPAQ-C asks each participant to fill in the frequency and duration of three different intensity levels of physical activity, including walking, moderate intensity, and vigorous intensity (26, 27), for at least 10 min at a time during the past 1 week. “Vigorous intensity,” refers to activities, such as heavy lifting, digging, aerobics, or fast bicycling (26, 27), that require hard physical effort and significantly increase the breathing rate of adults compared to their rest state. “Moderate intensity,” refers to activities, such as carrying light loads, bicycling at a regular pace, or doubles tennis (26, 27), that require moderate physical effort and somewhat increase the breathing rate of adults compared to their rest state. “Walking” refers to activities, such as walking at work and at home, walking from place to place, and any other walking that may be done solely for recreation, sport, exercise, or leisure (26, 27). Metabolic equivalent (METs) hours per week were calculated using the following formula: MET coefficients of activity (8.0, 4.0, and 3.3 for vigorous intensity, moderate intensity and walking, respectively) × duration (hours) × frequency (days); total weekly PA was calculated by summing the METs-hour/week score for different activities (26, 27).

### Statistical Analysis

All continuous variables and categorical variables are presented as mean ± SD or percentages (numbers). An analysis of variance or a chi-square-test was used to analyze the differences of baseline characteristics between handgrip strength categories.

We used the multivariate logistic regression analysis to examine the relationships between the change in handgrip strength at 1 year and the incidence risk of depressive symptoms during the follow-up period after adjustment for age (continuous variable) at baseline (Model 1). In Model 2, we adjusted for age (continuous variable), change in smoking status (increase, decrease, no change), change in drinking status (increase, decrease, no change), change in physical activity level (continuous variables), change in sleep quality (from good to bad, from bad to good, and no change), change in sleep duration (continuous variables) and change in BMI (continuous variable). *P* < 0.05 was considered significant in all two-sided tests. IBM SPSS Statistics 24.0 (IBM SPSS Inc., Chicago, IL, USA) was applied to do all statistical analyses.

## Results

A total of 599 participants aged 18.7 ± 1.0 years took part in the study. Table 1 shows the participants' baseline characteristics according to the categories of handgrip strength. Compared with participants in the categories of lowest handgrip strength, participants in the upper three categories tended to be younger (*P*-value: 0.012). There was a lower proportion of occasional and regular smoking participants (*P*-value: 0.040) among those with a higher handgrip strength level. The BMI differed significantly between handgrip strength categories (*P*-value: <0.001). Besides this, there were no significant differences observed across the handgrip strength categories.

**Table 1:** Participants' characteristics according to categories of handgrip strength^a^.

***N* = 599**	**Total**	**Group 1 (*n* = 99)**	**Group 2 (*n* = 222)**	**Group 3 (*n* = 185)**	**Group 4 (*n* = 93)**	***P*-value^b^**
Mean ± SD (kg)	17.7 ± 4.3	21.8 ± 1.3	25.8 ± 1.1	29.7 ± 1.2	34.8 ± 2.5	—
Age, years	18.7 ± 1.0	18.5 ± 0.8	18.7 ± 0.9	18.8 ± 1.0	18.6 ± 1.2	0.012
BMI, Kg/m^2^	20.2 ± 2.6	19.2 ± 2.2	20.1 ± 2.4	20.4 ± 2.6	21.4 ± 3.0	<0.001
Obesity categories						<0.001
Under weight, % (*n*)	27.5 (165)	43.4 (43)	30.2 (67)	24.9 (46)	9.7 (9)	
Normal weight, % (*n*)	59.4 (356)	50.5 (50)	59.0 (131)	60.5 (112)	67.7 (63)	
Overweight and obesity, % (*n*)	13.0 (78)	6.1 (6)	10.8 (24)	14.6 (27)	22.6 (21)	
Smoking status, %						0.040
Never	95.7 (573)	90.9 (90)	95.5 (212)	96.8 (179)	98.9 (92)	
Occasionally and regularly	4.3 (26)	9.1 (9)	4.5 (10)	3.2 (6)	1.1 (1)	
Drinking status, %						0.230
Never	50.3 (301)	55.6 (55)	45.0 (100)	53.5 (99)	50.5 (47)	
Occasionally and regularly	49.7 (298)	44.4 (44)	55.0 (122)	46.5 (86)	49.5 (46)	
PA, MET·h·week-1	54.9 ± 50.1	45.7 ± 36.4	50.8 ± 49.9	49.4 ± 40.2	42.8 ± 35.8	0.435
Sleep duration, hour	6.8 ± 0.9	6.9 ± 1.0	6.7 ± 0.9	6.8 ± 0.9	6.8 ± 1.0	0.467
Sleep quality (good), %	79.0 (473)	73.7 (73)	81.5 (181)	77.8 (144)	80.6 (75)	0.422

*PA, physical activity; BMI, body mass index; MET, metabolic equivalent; SDS, self-rating depression scale*.

a *Continuous variables are expressed as Mean ± SD and categorical variables are expressed as percentages (numbers)*.

b *Analysis of variance or chi-square-test, respectively*.

During the 1-year follow-up period, 64 of the 599 participants were classified as having depressive symptoms. Table 2 shows the relationship of baseline handgrip strength with the incidence risk of depressive symptoms during the 1-year follow-up period. There was no significant relationship between handgrip strength and the incidence risk of depressive symptoms after adjusting for relevant covariates.

**Table 2:** Adjusted odds ratios (95% confidence interval) of associations of baseline handgrip strength with depressive symptoms (SDS ≥ 50) during the 1-year follow-up period.

***N* = 599**	**Mean ± SD**	**Number of case**	**Model 1^a^**	**Model 2^b^**
Categories of handgrip strength	17.7 ± 4.3	—	—	—
Group 1 (*n* = 99)	21.8 ± 1.3	10	1.000 (reference)^c^	1.000 (reference)
Group 2 (*n* = 222)	25.8 ± 1.1	28	1.26 (0.59, 2.72)	1.53 (0.69, 3.39)
Group 3 (*n* = 185)	29.7 ± 1.2	20	1.04 (0.47, 2.35)	1.25 (0.54, 2.87)
Group 4 (*n* = 93)	34.8 ± 2.5	6	0.61 (0.21, 1.74)	0.83 (0.28, 2.49)
*P* for trend^d^	—	—	0.298	0.637

a *Model 1 adjusted for age (continuous variable) at baseline*.

b *Model 2 adjusted for Model 1 + smoking status (never, occasionally, or regularly), drinking status (never, occasionally, or regularly), physical activity level (continuous variable), sleep quality (good or not), sleep duration (6–8 h/d or <6 and >8 h/d) and BMI (continuous variable) at baseline*.

c *Adjusted data are expressed as odds ratio (95% confidence intervals)*.

d *P for trend were obtained using multivariate logistic regression analyses*.

**Figure 1:**
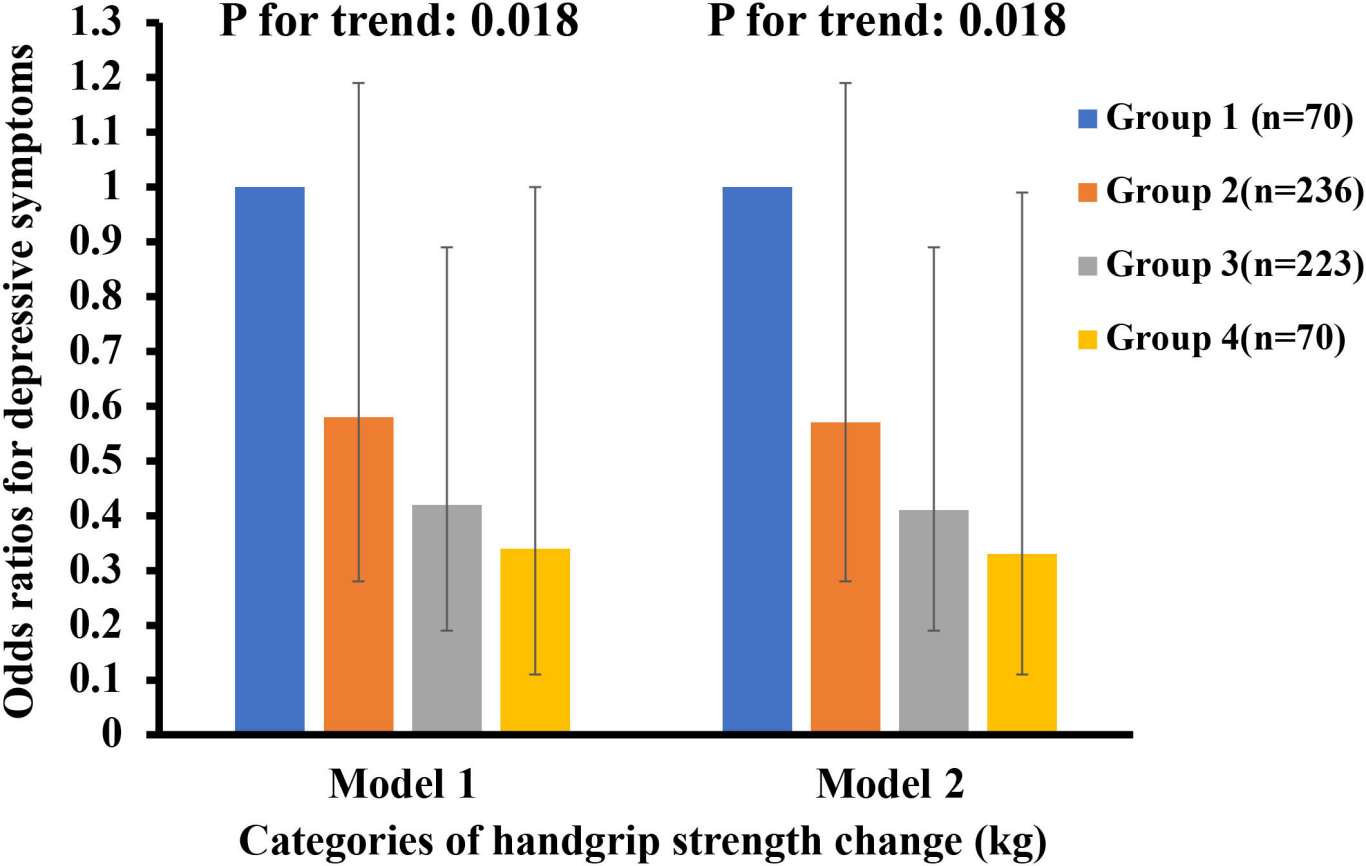
Adjusted odds ratios (95% confidence interval) of associations of categories of handgrip strength change (kg) with depressive symptoms (SDS ≥ 50) among Chinese college students. Model 1 adjusted for age (continuous variable); Model 2 adjusted for Model 1 + change of smoking status (increase, decrease, no change), change of drinking status (increase, decrease, no change), change of physical activity level (continuous variable), change of sleep quality (from good to bad, from bad to good, and no change), change of sleep duration (continuous variable) and change of BMI (continuous variable).

Association between the change in handgrip strength and the incidence risk of depressive symptoms is shown in Figure 1. After adjusting for potential confounders, there was a significant inverse association between change of handgrip strength and the incidence risk of depressive symptoms. The multivariable-adjusted odds ratio (95% CI) of depressive symptoms for the categories of handgrip strength change was 1.00 (reference) for group 1, 0.57 (0.28, 1.19) for group 2, 0.41 (0.19, 0.89) for group 3 and 0.33 (0.11, 0.99) for group 4 (*p* = 0.018).

Correlation coefficients between handgrip strength change and change of depressive symptoms scores are also presented in Table 3. Bivariate (*r* = −0.081) and partial (*r* = −0.088) correlations were significant.

**Table 3:** Correlations between change of handgrip strength and depressive symptoms score during 1-year.

**Variables**	**Change of handgrip strength**			
	Bivariate	*P*-value	Partial^a^	*P*-value
Change of depressive symptoms score	−0.081	0.049	−0.088	0.024

a *Partial correlations adjusted for age (continuous variable) at baseline, change of smoking status (increase, decrease, no change), change of drinking status (increase, decrease, no change), change of physical activity level (continuous variable), change of sleep quality (from good to bad, from bad to good, and no change), change of sleep duration (continuous variable)and change of BMI (continuous variable) during 1-year*.

## Discussion

In this 1-year prospective cohort study, we analyzed the association between change in handgrip strength and the incidence risk of depressive symptoms. This study showed that an increase in the level of handgrip strength, over a 1-year period, was associated with a lower risk of depressive symptoms after adjusting for potential confounders.

In two large scale cross-sectional studies, there were significant associations between a lower baseline handgrip strength level and higher risk of depressive symptoms among an elderly population aged 50 years and over (13–15). Similarly, several authors also conducted an elderly population-based prospective cohort study and demonstrated that baseline handgrip strength level is protective against development of depressive symptoms over time (16–19). However, these studies were limited to an elderly population and only investigated baseline handgrip strength level, whether dynamic changes in handgrip strength level have a negative or positive effect on risk of depressive symptoms is unclear. We examined for the first time the association between dynamic changes in handgrip str-ength level and the risk of depressive symptoms among Chinese college students in a prospective cohort study.

We explored several possible mechanisms. First, skeletal muscle, which acts as an endocrine organ, can secrete and produce numerous proinflammatory cytokines, such as IL-6, IL-8, and IL-15 (10). In two younger adults-based studies, weaker skeletal muscle strength has been suggested to be associated with increased serum proinflammatory cytokines (11, 12). Meanwhile, increased inflammatory cytokines secretion can alter neurotransmission (5), hippocampal neurogenesis (6), and stress-related HPA axis (7) and sympathetic system activation (8), which can cause changes in structure and function of the brain, and subsequent development of depression (9).

Second, oxidative stress may mediate the association between handgrip strength change and risk of depressive symptoms. It is well-known that oxidative stress reflects a disequilibrium status between status of prooxidant and antioxidant reactions in living organisms. Depression is characterized by an elevation of immune activation (a generator of reactive oxygen species) (28), and activated immune cells may cause excessive production of reactive oxygen species, which results in an increase in malondialdehyde levels (a generator of oxidative stress) (29). Conversely, in a randomized controlled trial, researchers explored the effect of resistant exercise on the generator of oxidative stress and anti-oxidant capacity in young women aged 18–25 years, and found that long-term resistant exercise induced decreased level of malondialdehyde, and elevated total anti-oxidant capacity level (30). As skeletal muscle strength may be effectively improved by muscle-strengthening activities such as resistance training, we reasonably speculate that oxidative stress could mediate the inverse association between handgrip strength level and risk of depressive symptoms.

In this study, we also adjusted for several relevant covariates, including age, smoking status, drinking status, sleep quality, sleep duration, and BMI. A meta-analysis of longitudinal studies showed that obese adolescents had a higher risk of depression (31). Meanwhile, handgrip strength increased with weight status gain (32). However, after additionally adjustment for BMI, the association between handgrip strength change and risk of depressive symptoms remained significant. Lifestyle factors, including smoking (33) and drinking habits (34) could also confound the association between handgrip strength change and risk of depressive symptoms. Additionally, this study also found that change in sleep duration and quality was negatively associated with change in depressive symptoms score (Supplementary Table 4). However, a significant association between handgrip strength and depressive symptoms still remained after adjustment for change in sleep duration and quality. We concluded that there is a independent association between handgrip strength and depressive symptoms.

### Limitations

Several limitations should be mentioned in our study. First, since this study is a young population-based observational study, we cannot establish whether or not there is a causal association between handgrip strength and depressive symptoms. Second, the participants of this study were limited to Chinese female college students, and whether the above-mentioned association also exists in Chinese other college students remains unknown. Third, since we used SDS to investigate depressive symptoms, it is unclear whether these college students with depressive symptoms have clinical depression. Fourth, this study did not measure skeletal muscle mass, and therefore, change of skeletal muscle mass could confound the association between handgrip strength change and risk of depressive symptom. Fifth, we did not collect information on intensity and type of physical activity because IPAQ only provides an estimate of physical activity, but not of type and intensity of physical activity (35). WHO recommends that adults aged 18–64 years should do activities that strengthen muscle for 2 or more days per week for at least 150 min of moderate-intensity aerobic physical activity (or 75 min of vigorous-intensity aerobic physical activity), to improve muscle strength (36). Future studies should investigate whether the relationship between handgrip strength change and risk of depressive symptoms will be attenuated when adjusting for type and intensity of physical activity. Sixth, dietary intake and social support could confound the association between handgrip strength change and risk of depressive symptoms. It is possible that individuals with a higher level of handgrip strength may have healthier dietary patterns (37). Furthermore, a Chinese cross-sectional study indicated that college students who obtained greater social support have lower risk of depression (38). Unfortunately, we did not collect data on variables such as dietary intake and social support. Therefore, the relationship between handgrip strength change and risk of depressive symptoms may have been overestimated. Finally, exclusion of 27% (296/1,094) of participants with missing information at baseline and follow-up period might have created a bias in our results. The association between the change in handgrip strength and the incidence risk of depressive symptoms could have been underestimated or overestimated.

## Conclusions

This study indicates that handgrip strength change over 1-year period is negatively associated with risk of depressive symptoms among Chinese female college students. Further studies should be warranted to confirm the causality of this relationship using randomized controlled trial.

## Data Availability Statement

The raw data supporting the conclusions of this article will be made available by the authors, without undue reservation.

## Ethics Statement

The studies involving human participants were reviewed and approved by Institutional Review Board of the College of Physical Education of Southwest University. Written informed consent to participate in this study was provided by all college freshmen aged ≥16 years or from the legal guardians of participants aged <16 years.

## Author Contributions

ZR and FZ: conceptualization and methodology. ZR and JC: formal analysis. JC, FZ, and ZR: data curation. JC: writing—original draft preparation, review, and editing. All authors: have read and agreed to the published version of the manuscript.

## Conflict of Interest

The authors declare that the research was conducted in the absence of any commercial or financial relationships that could be construed as a potential conflict of interest.
