# Supplementary material for: Association Between Changes in Muscle Strength and Risk of Depressive Symptoms Among Chinese Female College Students: A Prospective Cohort Study
Source: Front Public Health. 2021 Apr 8;9:616750. doi: 10.3389/fpubh.2021.616750 (PMC8060500; doi:10.3389/fpubh.2021.616750)
Supplement: Supplementary file 1 [file Data_Sheet_1.docx]

**Table S1. Adjusted associations of change of handgrip strength (continuous variable) with depressive symptoms (SDS ≥ 50) during the one-year follow-up period.**

| N = 599 | B (95% CI) | β | P value ^c^ |
| --- | --- | --- | --- |
| Model 1 ^a^ | 0.94 (0.88, 1.01) | -0.06 | 0.093 |
| Model 2 ^b^ | 0.94 (0.88, 1.01) | -0.06 | 0.083 |

^a^ Model 1 adjusted for age (continuous variable) at baseline;

^b^ Model 2 adjusted for Model1 + change of smoking status (increase, decrease, no change), change of drinking status (increase, decrease, no change), change of physical activity level (continuous variable), change of sleep quality (from good to bad, from bad to good, and no change), change of sleep duration (continuous variable) and change of BMI (continuous variable).

^c^ P for trend were obtained using multivariate logistic regression analyses.

$n=\left( \frac{u_{\alpha}\sqrt{2PQ}+u_{\beta}\sqrt{P_{1}\left( 1-P_{1} \right)+P_{2}\left( 1-P_{2} \right)}}{P_{1}-P_{2}} \right)^{2}$

n= 119

119 participants would sufficiently investigate the associations of change of handgrip strength and risk of depressive symptoms.

Assuming a=0.05 and the power of the study (1-$\beta$) = 0.90, 119 participants would be needed to detect a difference of 2 standard Self-rating Depression Scale (SDS) and Self-rating Anxiety Scale (SAS) scores between high PA and low PA group [19,20]

**Table S2**. Adjusted associations of change of handgrip strength with depressive symptoms (SDS ≥ 50) during the one-year follow-up period.

| N = 599 | Categories of handgrip strength change | | | | P for trend ^h^ |
| --- | --- | --- | --- | --- | --- |
|  | Group 1 (n =70) | Group 2 (n =236) | Group 3 (n =223) | Group 4 (n =70) |  |
| Mean±SD | -6.0±3.0 | -1.4±1.0 | 1.8±1.1 | 6.4±3.3 | - |
| Number of Case | 13 | 27 | 19 | 5 | - |
| Model 1 | 1.000 (reference) | 0.58 (0.28, 1.19) | 0.42 (0.19, 0.89) ^i^ | 0.34 (0.11, 1.00) | 0.018 |
| Model 2 | 1.000 (reference) | 0.56 (0.27, 1.16) | 0.40 (0.19, 0.86) ^i^ | 0.33 (0.11, 0.98) ^i^ | 0.015 |
| Model 3 | 1.000 (reference) | 0.57 (0.28, 1.17) | 0.41 (0.19, 0.88) ^i^ | 0.34 (0.11, 1.01) | 0.018 |
| Model 4 | 1.000 (reference) | 0.57 (0.28, 1.17) | 0.41 (0.19, 0.88) ^i^ | 0.34 (0.11, 1.02) | 0.019 |
| Model 5 | 1.000 (reference) | 0.56 (0.27, 1.16) | 0.40 (0.19, 0.87) ^i^ | 0.34 (0.11, 1.00) | 0.017 |
| Model 6 | 1.000 (reference) | 0.56 (0.27, 1.16) | 0.41 (0.19, 0.89) | 0.34 (0.11, 1.01) | 0.019 |
| Model 7 | 1.000 (reference) | 0.57 (0.28, 1.17) | 0.41 (0.19, 0.88) | 0.34 (0.11, 1.00) | 0.018 |

^a^ Model 1 adjusted for age (continuous variable) at baseline;

^b^ Model 2 adjusted for change of smoking status (increase, decrease, no change)

^c^ Model 3 adjusted for change of drinking status (increase, decrease, no change)

^d^ Model 4 adjusted for change of physical activity level (continuous variable)

^e^ Model 5 adjusted for change of sleep quality (from good to bad, from bad to good, and no change)

^f^ Model 6 adjusted for change of sleep duration (continuous variable)

^g^ Model 7 adjusted for change of BMI (continuous variable)

^h^ P for trend were obtained using multivariate logistic regression analyses.

^i^ Significantly different from group 1, P<0.05.

**Table S3**. Associations of change of sleep duration and quality with change of depressive symptoms score during the one-year follow-up period.

| N = 599 | Categories of sleep duration change ^1^ | | | | P value ^2^ |
| --- | --- | --- | --- | --- | --- |
|  | Group 1 (n =54) | Group 2 (n =145) | Group 3 (n =346) | Group 4 (n =54) |  |
| Change of depressive symptoms | 1.3±8.6 | 0.2±8.9 | -3.0±8.1 | -2.1±8.4 | <0.001 |
|  | Categories of sleep quality change | | | |  |
|  | Group 1 (n =55) | Group 2 (n =457) | Group 3 (n =87) |  |  |
| Change of depressive symptoms | 1.9±8.5 | -1.8±8.4 | -3.9±8.2 |  | <0.001 |

^1^ One-year change in sleep duration was divided into 4 categories according to ± 1SD: group 1 (<-0.5), group 2 (0, 0.5), group 3 (1, 2), and group 4 (>2.5)

^2^ P value were obtained using analysis of variance.
